# Association between Adjusted Handgrip Strength and Metabolic Syndrome in Arab Men

**DOI:** 10.3390/ijerph182010898

**Published:** 2021-10-17

**Authors:** Shaea Alkahtani

**Affiliations:** Department of Exercise Physiology, College of Sport Sciences and Physical Activity, King Saud University, P.O. Box 2454, Riyadh 11451, Saudi Arabia; shalkahtani@ksu.edu.sa or shayaedris22@gmail.com; Tel.: +966-118-063-288 or +966-551-504-004; Fax: +966-118-063-370

**Keywords:** appendicular lean mass, handgrip strength, body fat, body weight, body mass index

## Abstract

This cross-sectional study determined the association between handgrip strength (HGS) and metabolic syndrome (MetS) in Arab men. Furthermore, HGS and adjusted HGS, relative to body composition components including body mass index (BMI), body weight, and body fat percentage (%Fat), were examined in predicting MetS. Methods: In this study, 854 men participated in and completed all tests (age, 39.7 ± 15.2 years; BMI, 28.4 ± 5.2 kg/m^2^; %Fat, 26.6% ± 7.1%). Body composition and HGS were measured using a body impedance analyzer and a manual spring-type dynamometer, respectively. About 10 cc of venous blood was drawn once after overnight fasting and analyzed using the colorimetric method. MetS included waist circumference (WC), triglycerides (TG), high-density lipoprotein cholesterol (HDL-C), blood pressure (BP), and fasting glucose were defined for the current specific population. Results: The receiver operating characteristics curve (ROC curve) showed an area under the curve (AUC) of HGS = 0.54, and 0.70 for HGS/%Fat. Linear regression analysis showed that the R^2^ values for all three models were low in predicting MetS and its components. Lastly, the odds ratio of adjusted HGS showed that there were significant differences between all quartiles of MetS compared with the reference quartile (Q1), whereas HGS alone did not show such differences. A significant difference between the quartiles of HGS and adjusted HGS was observed in Q4 for glucose, and significant differences were also found from Q2 for hypertension in terms of the HGS and adjusted HGS. Conclusion: HGS could have protective potential for increased levels of glucose and systolic blood pressure, and using adjusted HGS rather than HGS alone is recommended for the association of MetS in Arab men.

## 1. Introduction

In 1989, sarcopenia was initially defined by Irwin Rosenberg as an age-related decrease in muscle mass [1]. It was recognized as an independent disease in 2016 with the code ICD-10-CM [2]. It has a standard reference of conceptual stages as a decrease in muscle mass (presarcopenia), a decline in muscle strength (sarcopenia), and a decline in muscle power (severe sarcopenia) [3]. Recently, the European Working Group on Sarcopenia in Older People revised the definition of sarcopenia (EWGSOP2), making muscle strength the first-line measure of diagnostic sarcopenia, which is called probable sarcopenia [4]. Several studies have used body weight and composition, including muscle mass, to explain changes in energy metabolism, glucose disposal, and risk of cardiometabolic diseases [5,6,7]. The ability of muscle strength compared with muscle mass to predict and explain health parameters is important in practical clinics, but it is still uncertain. In a cross-sectional sarcopenia study in Saudi Arabia, multiple regression analyses of three parameters (age and anthropometric measurements, body composition, and nutrient intake) using either the appendicular lean body mass divided by the height squared (ALM/h^2^) or the handgrip strength (HGS) explained variations (R^2^) in age and anthropometric measurements by 0.86 and 0.36, body composition by 0.71 and 0.34, and nutrient intake by 0.45 and 0.14, respectively [8]. Whether muscle strength can explain changes in metabolic markers requires further investigation, particularly in the Arab world.

Body mass is strongly correlated with muscle strength [9], which implies that heavier people are stronger [10]. The existence of muscle weakness and low body weight, as well as their association with chronic diseases, has been observed. For example, in a 30-year follow-up study involving disease-free healthy men, body mass index (BMI) showed a minimal effect on mortality within HGS tertiles, whereas mortality risk decreased as HGS increased in each BMI category [11]. However, a systematic review and meta-analysis have shown that grade 1 obesity is not associated with higher mortality; being overweight is even associated with lower all-cause mortality [12]. Thus, distinguishing the effects of body mass from those of body composition on health aspects is important, and variations in body composition should be considered when determining the association between body mass and health aspects. Likewise, different indices of body composition may have different associations with muscle strength. For example, in Saudi men, differences between low and high HGS tertiles were significant in body weight and muscle mass, but they did not reach significance in BMI and fat mass [13]. Note that the latest European consensus on sarcopenia [4] considered the adjusted muscle quantity relative to the body composition, but without emphasis.

Metabolic syndrome (MetS) has been defined by the leading scientific organizations in the world, including the World Health Organization (WHO) [14], the International Diabetes Federation (IDF) [15], the National Cholesterol Education Program (NCEP) Adult Treatment Panel III (ATP III) [16], the American Heart Association (AHA), and the National Heart, Lung, and Blood Institute (NHLBI) [17]. These organizations agree that MetS is a cluster of cardiometabolic markers including obesity, dyslipidemia, hypertension, and elevated blood glucose. Between the different definitions, there are minor differences in the selection of three markers and cutoff points. According to a Joint Interim Statement from the representatives of the leading international organizations in the world [18], a country-specific definition of WC has been called for. For example, a country-specific WC cutoff of ≥90 cm has been used in a study in Korea [19], whereas a cutoff of ≥94 cm has been suggested as an appropriate definition for Middle East and Mediterranean males based on this consensus [18].

MetS has been linked to low muscle mass [7] and low handgrip strength [20], and some studies have suggested adjusting the HGS (i.e., relative to body composition measurements) to link with MetS. For example, the Sixth Korea National Health and Nutrition Examination found that HGS was not related to MetS, whereas adjusted HGS relative to BMI showed a significant dose–response relationship in elderly participants, and both measures showed a similar correlation with the quality of life [19]. HGS relative to body weight and BMI, but not HGS alone, showed a significant inverse association with MetS and its components with a cluster of factors, including smoking and physical activity, in old Singaporean adults [21]. The Korean Longitudinal Study of Ageing has examined the association of HGS and adjusted HGS relative to BMI with hypertension [22]. HGS and adjusted HGS were significantly associated with hypertension, and the odds ratio (OR) of adjusted HGS from low to high was more predictive of hypertension, although both had the same trend. Based on a Taiwanese cohort cross-sectional study, calculating adjusted HGS by summing both HGS scores divided by BMI demonstrated several cardiometabolic risk factors, particularly in men [23]. While the link between adjusted HGS and MetS has been suggested in some countries, similar data from Saudi Arabia and the Arab world are warranted. Therefore, the current study hypothesizes that HGS adjusted to body composition is better than HGS alone in predicting MetS in Arab men.

## 2. Materials and Methods

### 2.1. Participant Characteristics

In this study, announcements were posted through the university, social media, and community development commissions of Riyadh’s districts. Individuals who expressed their interest in participating in the study were recorded as samples of the study. In total, 1046 men were assessed for eligibility and 60 participants were excluded because they did not meet the inclusion criteria (mostly because they had ongoing metabolic treatments and/or uncertain health conditions). Then, 986 men were recruited to participate in the study, and 108 withdrew because they did not respond after the first communication. Data for 878 men were collected and analyzed. Data of HGS for nine participants were missing, and blood samples for 15 participants were not included because of coding and/or analysis errors. Data for 854 men who participated and completed all tests (age, 39.7 ± 15.2 years; BMI, 28.4 ± 5.2 kg/m^2^; body fat percentage (%Fat), 26.6% ± 7.1%) were analyzed. The participants were healthy men, predominantly Saudis, while 184 participants were from the Middle East and lived in Saudi Arabia. Inclusion criteria included all men above 18 years old regardless of their obesity degree or recreational physical activity level. Exclusion criteria included official athletes in sport teams, individuals with a diagnosed illness affecting mobility, those with uncontrolled and/or severe metabolic disease (e.g., type 1 diabetics with uncontrolled blood sugar and hypertensives with uncontrolled blood pressure), and those with uncertain health conditions, chronic diseases, or ongoing medical treatment (e.g., cardiac, kidney, or glandular treatment).

### 2.2. Study Procedure

This cross-sectional study was conducted in two stages between 2016 and 2018. Data were collected at two locations: the College of Sport Sciences and Physical Activity at King Saud University (KSU) and at community development commissions in Riyadh districts, which were air-conditioned at 21–22 °C. All participants provided written informed consent. The institutional review board of KSU approved the study protocol (IRB no. E-18-3381).

Participants were asked to maintain their lifestyle prior to the measurements (i.e., habitual sleep, diet, and physical activity). On the day prior to assessment, participants were asked to avoid extensive physical work and the over-consumption of caffeine and salty food. Data were collected in the morning after overnight fasting. The study procedure was previously explained to the participants, and upon their arrival at the data collection center, they started the measurements of study variables, which were arranged and ordered in a sequence. Measures began with anthropometry, body composition, and blood sample collection, and finished with the measuring of HGS. The following sections will illustrate the instruments used, management of data collection, and acquisition of study variables in greater detail.

### 2.3. Measures of Body Composition and HGS

Anthropometric parameters including height, weight, and waist circumference (WC) were measured to the nearest 0.1 cm using a stadiometer (seca 213; seca Gmbh & Co. KG., Hamburg, Germany), a digital scale (PD100 ProDoc; Cardinal/Detecto, Webb City, MO, USA), and a measuring tape at the umbilicus. Body composition was measured using a multi-frequency segmental body composition analyzer (Tanita MC-980MA; Tanita Corporation, Tokyo, Japan) using currents of 1000 kHz. The output of the measure included information on several body composition elements, including muscle and fat mass.

The HGS of the dominant hand was measured using a manual spring-type dynamometer (Baseline^®^ Smedley spring-type dynamometer, Fabrication Enterprises, Inc., White Plains, NY, USA). The handle grip was adjusted before the performance to a comfortable position for the individual. The test was performed while participants were standing with the elbow at full extension and shoulder at a natural position at zero degrees. Two trials were performed with 60-s intervals, and the best/highest trial was recorded in kg. Participants were instructed to squeeze the handle as hard as they could for as long as they could until the instructor said to stop (when the needle stopped rising); verbal encouragement was provided during the tests [24].

#### Data Management of Body Composition

The participants were divided into three subgroups based on HGS. The average HGS for Saudi males (42 kg) [25] and the cutoff for low HGS in males based on the EWGSOP2 definition (27 kg) were used to classify the participants into subgroups (low ≤ 27 kg, medium > 27 kg–42 kg, high > 42 kg). Moreover, HGS was presented in the results as an absolute value in kg, and was also presented in relation to body composition (adjusted HGS) as follows: HGS adjusted to body weight (HGS/weight), HGS adjusted to BMI (HGS/BMI), and HGS adjusted to the fat percentage (HGS/%Fat).

### 2.4. Measures of MetS

About 10 cc of fasting venous blood was drawn from the antecubital vein by a well-trained phlebotomist after overnight fasting for at least 10 h. Blood samples were processed for separation of serum samples on the same day of collection. The remaining blood and serum samples were transported to King Khalid University Hospital (KKUH, KSU, Riyadh, Saudi Arabia) in specialized containers for biochemical analyses and storage at −80 °C. The fasting blood profile including TG, high-density lipoprotein cholesterol (HDL-C), and glucose was determined by colorimetric methods using the Dimension^®^ EXL^TM^ with an LM Integrated Chemistry System (Siemens Healthineers, Erlangen, Germany), and following a standard operating procedure at KKUH. All the outcomes were checked for normality and accuracy and recorded in mg/dL.

Participants were considered to have MetS if they had three or more of the following components: WC ≥ 94 cm, TG ≥ 150 mg/dL (1.7 mmol/L), HDL-C < 40 mg/dL (1.0 mmol/L), blood pressure (BP) ≥ 130/85 mmHg, and fasting glucose ≥ 100 mg/dL (5.6 mmol/L) [18]. In addition, data on the current medical treatment, pharmacological use, and previous diagnostic diseases were obtained.

#### Data Management of MetS

The relationships between HGS and adjusted HGS (HGS/weight, HGS/BMI, HGS/%Fat) with MetS and its components were examined in the current study using three methods: receiver operating characteristic curve (ROC), predictive model, and odds ratio (OR). In the ROC analysis, the threshold between the true positive rate and the false positive rate (sensitivity and specificity) was determined at 45 degrees, and data were plotted on a diagram to show the AUC of HGS and adjusted HGS. In our predictive model, the independent variables included age, HGS, and a body composition variable (weight, BMI, %Fat), and a dependent variable was one of the MetS components. Lastly, in the OR analysis, participants were divided to four quartiles. The reference group (OR = 1) was the lowest quartile of HGS and adjusted HGS (e.g., HGS quartiles were (Q1 <35 kg), Q2 (35–40 kg), Q3 (40.1–46 kg), and Q4 (>46 kg)). Thus, an OR of 1 meant there was no association between exposure and outcome (similar odds of exposure among patients and controls), whereas an OR of 0.5 indicated that the exposed group of HGS and adjusted HGS had half (50%) the odds of developing the disease (MetS and its components) when compared to the unexposed group [26,27]. So, an OR below 1 meant a protective role (a lower probability/chance) of HGS and adjusted HGS in developing MetS and its components.

### 2.5. Statistical Analysis

All the statistical analyses were performed using Statistical Package for the Social Sciences (version 22; IBM Corp., Armonk, NY, USA). Continuous data are presented as the mean ± standard deviation for normal variables, and non-Gaussian variables are presented as median (first and third) percentiles. Categorical data are presented as frequencies (N). The normality of the distribution of all continuous variables was checked using the Kolmogorov–Smirnov test. Non-Gaussian variables were log-transformed before the parametric analysis. The independent *t*-test and Mann–Whitney U-test were used to compare the mean and median differences between Gaussian and non-Gaussian variables. Analysis of variance, the Kruskal–Wallis H test, and post hoc Bonferroni analysis were performed to compare more than two groups. Binary logistic and linear regressions were performed to identify independent predictors of MetS and its components. Pearson’s and Spearman’s correlation analyses were performed to determine the correlations between independent predictors and MetS components. A ROC curve was drawn up for MetS with reference to control subjects for HGS, HGS/BMI, HGS/weight, and HGS/%Fat. *p*-values of less than 0.05 were used to denote statistical significance.

## 3. Results

### 3.1. Description of HGS Categories

The participants were divided into three subgroups based on HGS. The *p*-values and the significance of the differences in body composition, metabolic markers, and HGS parameters between these subgroups are shown in Table 1.

### 3.2. True-Positive and False-Negative Rates of Adjusted HGS Test Performance

**Figure 1:**
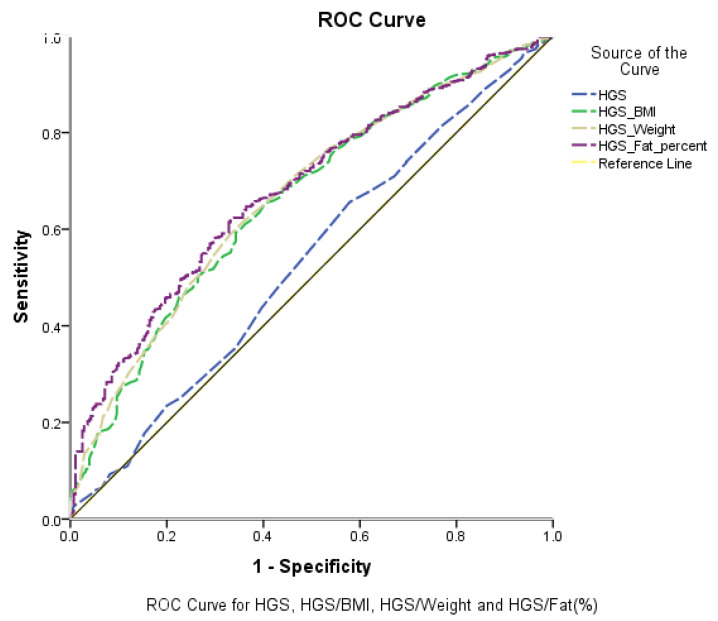
Receiver operating characteristic curve (ROC curve) for handgrip strength (HGS), handgrip strength adjusted to body mass index (HGS/BMI), handgrip strength adjusted to weight (HGS/weight), and handgrip strength adjusted to body fat percentage (HGS/%Fat) for metabolic syndrome (MetS).

The correlations of HGS and adjusted HGS with anthropometric parameters and body composition (HGS/BMI, HGS/weight, and HGS/%Fat) were positive and large (r = 0.7, 0.61, and 0.52, respectively; *p* < 0.01). Table 2 and Figure 1 show the ROC curve for HGS and adjusted HGS with MetS and its components. HGS/%Fat was the highest significant predictor of MetS (AUC = 0.70, *p* ≤ 0.01) and its components (AUC = 0.59–0.83, *p* ≤ 0.01), whereas HGS had the lowest predictive power of MetS (AUC = 0.54, *p* > 0.05) and its components (AUC = 0.49–0.54, *p* > 0.05).

### 3.3. Predictive Models of MetS Components

A linear regression analysis of dependent metabolic components (WC, SBP, DBP, glucose, HDL-C, and TG) with independent predictors including age, HGS, and one parameter of body composition (weight, BMI, and %Fat) was performed in models 1, 2, and 3, respectively (Table 3).

### 3.4. Association between Adjusted HGS and Risk of MetS and Its Components

Table 4 shows the OR (95% confidence interval (CI)) for MetS with HGS, HGS/BMI, HGS/weight, and HGS/%Fat based on quartiles. No significant association was observed between HGS (reference Q1 < 35 kg) and Q2 (35–40 kg), Q3 (40.1–46 kg) or Q4 (>46 kg), respectively. Furthermore, the adjusted HGS relative to BMI, weight, and %Fat was observed in all quartiles to have significant relationships with MetS for all parameters (HGS/BMI, HGS/weight, and HGS/%Fat).

Table 5 shows the ORs for the MetS components with the study variables. The ORs in Q4 of adjusted HGS (HGS/BMI, HGS/weight, and HGS/%Fat) with all MetS components and in HGS with glucose were significant. The value of the OR constantly decreased through the quartiles (Q2–Q4) in all adjusted HGS analyses. Most quartiles of HGS and adjusted HGS in association with SBP were significant.

## 4. Discussion

The current study examined the association between HGS and adjusted HGS with MetS and its components. Table 1 shows that, while the BMI significantly increased as HGS increased, the opposite relationship was observed between %Fat and HGS. This could likely affect the relationship between HGS/BMI and HGS/%Fat with metabolic health. The ROC curve (Figure 1) showed that HGS was the lowest, with AUC = 0.54, whereas HGS/%Fat was the highest at 0.70. Although Table 1 showed significant differences between low HGS (<27 kg) and medium-to-high HGS in BP and HDL-C, ROC analysis (Figure 1 and Table 2) demonstrated that HGS is not a good classifier of individuals with MetS, and none of its relationships with MetS components were significant (*p* > 0.05). The linear regression analysis (Table 3) showed that the R^2^ values for all three models ranged between 0.02 and 0.13 for the dependent variables (DBP, glucose, HDL, TG), between 0.18 and 0.19 for SPB, and between 0.39 and 0.42 for WC. Although the prediction of WC can be considered moderate [28], it should be noted that the independent body composition variables (weight, BMI, %Fat) in the three models explained the variation in the dependent body composition variable (WC). Lastly, based on adjusted HGS quartiles, there were significant differences between all quartiles of MetS compared with the reference quartile (Q1), whereas HGS alone did not show such differences (Table 4). It should be noted that the ORs for body composition parameters (weight, BMI, and %Fat) in association with MetS were large (2.00–11.00) and significant in all quartiles (data not included in Table 4). As shown in Table 5, a significant difference between the quartiles of HGS and adjusted HGS was observed in Q4 for glucose, and significant differences were also found from Q2 for hypertension in HGS and adjusted HGS. These outcomes suggest that HGS could have protective potential of increased glucose and BP, and adjusted HGS is recommended when predicting MetS in Arab men.

The differences between body composition parameters (BMI and %Fat) through HGS categories (Table 1) suggest that further consideration is required of the appropriate use of each parameter. Liao et al. [29] found a strong association between body mass and HGS through significant differences in HGS between low-, medium-, and high-BMI subgroups (26.80 ± 5.83 kg, 34.55 ± 7.84 kg, and 42.30 ± 5.35 kg, respectively). In our previous study involving Saudi men [25], HGS was 40.1 ± 6.9 kg for individuals with a BMI below 24 kg/m^2^, 43.1 ± 7.5 kg for participants with a BMI between 24.1 and 28 kg/m^2^, and 44.1 kg for participants with a BMI greater than 28 kg/m^2^. Alternatively, HGS was negatively associated with %Fat, particularly among Asian subjects who had a lower total muscle mass to height ratio and ALM/BMI ratio than other ethnicities [30]. Moderate inverse correlations were observed between HGS and %Fat in patients with type 2 diabetes mellitus [31]. Using a regression model, in obese participants, a higher weight positively affected strength parameters, but an increased fat mass index negatively affected strength parameters; no correlation was found between BMI and HGS. This means that fat distribution, rather than BMI, should be considered in assessing strength [32].

As noted previously, we have observed that HGS, independent of body composition, could have the potential to prevent diabetes and hypertension. HGS was suggested as a good marker for the fitness level and overall health, which could attenuate adiposity and metabolic risk factors. For example, some studies have found an inverse association between muscle strength and diabetes, hypertension [33], and MetS [34,35]. Overweight individuals with high HGS had a lower metabolic risk score, %Fat, and visceral fat than their peers who had low HGS, and moderately obese individuals with high HGS had lower visceral fat than their peers who had low HGS [36]. Moreover, hyperglycemia and excess central adiposity are two independent factors that accelerate the decline in muscle mass and strength. Data showed that abdominal obesity was inversely associated with fasting glucose, and fasting glucose was inversely associated with HGS; thus, the fasting glucose level mediates the relationship between abdominal obesity and HGS [37]. Thus, causality between HGS and cardiometabolic markers, as well as their interaction with body composition, requires further prospective studies.

The limitations of this study include the fact that there are no significant differences between HGS subgroups in fasting glucose and other metabolic markers, while the mean values of fasting glucose are similar in all groups; however, the ages of subgroups are significantly different (Table 1). This could be attributed to a bias in participant selection. The current study attracted healthy individuals rather than individuals with metabolic illnesses. This should be taken into consideration when referring to the current outcomes. Moreover, the average age of participants in the current study was 39, and it was conducted on an entirely male population. In the literature, diabetes was inversely related to HGS in younger women and men, but this was not found in elderly individuals, and HGS was associated with DBP in elderly men, whereas HGS was associated with SBP in young women; however, other markers did not show any associations [38]. In another study, moderate inverse correlations were observed between HGS and HDL-C in participants with type 2 diabetes mellitus, and a moderate inverse correlation was found between HGS and HbA1c% in healthy participants [31]. Future studies that are interested in sarcopenia should examine the association between HGS and adjusted HGS with MetS in the older population of Arab men and women.

## 5. Conclusions

HGS could represent a potential means of identifying those at risk of increased levels of glucose and SBP, so their levels can be prevented from rising; it is suggested that adjusted HGS rather than HGS alone should be used for the prediction of MetS in Arab men.

## Figures and Tables

**Table 1:** Differences in handgrip strength between subgroups based on body composition, metabolic markers, and handgrip strength parameters.

Parameters	<27kg HGS	27–42 kg HGS	>42kg HGS	*p*-Value
N	60	434	360	
HGS (kg)	23.0 ± 3.3	36.5 ± 3.8 ^A^	48.5 ± 4.4 ^AB^	<0.001
HGS/BMI	0.90 ± 0.2	1.35 ± 0.3 ^A^	1.70 ± 0.3 ^AB^	<0.001
HGS/weight	0.33 ± 0.08	0.48 ± 0.09 ^A^	0.57 ± 0.11 ^AB^	<0.001
HGS/%Fat	0.93 ± 0.6	1.50 ± 0.5 ^A^	2.0 ± 0.6 ^AB^	<0.001
Age (years)	57.8 ± 17.4	40.8 ± 15.6^A^	35.5 ± 11.8^AB^	<0.001
Height (cm)	165.8 ± 6.4	168.4 ± 6.4 ^A^	173.1 ± 6.1 ^AB^	<0.001
Weight (kg)	76.2 ± 17.8	79.3 ± 15.6	87.0 ± 16.1 ^AB^	<0.001
BMI (kg/m^2^)	27.3 ± 5.4	27.9 ± 5.1	29.1 ± 5.1 ^AB^	0.002
Fat component (%)	28.8 ± 8.3	26.5 ± 7.1	26.3 ± 6.8 ^A^	0.037
Fat free mass (kg)	52.7 ± 8.8	57.4 ± 7.6 ^A^	63.4 ± 7.4 ^AB^	<0.001
Muscle mass (kg)	50.5 ± 9.1	54.6 ± 7.3 ^A^	60.2 ± 7.0 ^AB^	<0.001
Waist circumference (cm)	90.8 ± 23.1	90.8 ± 19.4	92.9 ± 18.2	0.271
Systolic BP (SBP) (mmHg)	127.1 ± 19.5	119.6 ± 15.6 ^A^	119.7 ± 13.9 ^A^	0.002
Diastolic BP (DBP) (mmHg)	72.7 ± 10.8	75.3 ± 10.9	76.5 ± 10.2 ^A^	0.026
Glucose (mg/dL)	94.6 (82.4–157.6)	94.8 (82.7–136.0)	94.2 (83.6–111.9)	0.498
Total cholesterol (mg/dL)	181.5 ± 58.2	177.9 ± 56.9	184.9 ± 54.3	0.218
HDL cholesterol (mg/dL)	39.6 ± 10.8	43.9 ± 10.8 ^A^	42.9 ± 11.4	0.021
LDL cholesterol (mg/dL)	115.6 ± 54.7	109.9 ± 55.4	116.3 ± 50.9	0.254
TG (mg/dL)	106.3 (84.0–177.3)	103.9 (76.9–152)	102.8 (77.9–147.1)	0.477

**Note**: Data are presented as mean ± standard deviation for continuous normal variables and medians (25th percentile and 75th percentile) for continuous non-normally distributed variables; *p*-values are significant at the 0.05 and 0.01 levels. Superscripts A and B represent significance for <27 kg HGS and 27–42 kg HGS, respectively. HGS: handgrip strength; BMI: body mass index; BP: blood pressure; HDL: high-density lipoprotein; LDL: low-density lipoprotein; TG: triglycerides.

**Table 2:** Receiver operating characteristic curve for handgrip strength, handgrip strength relative to body mass index, handgrip strength relative to weight, and handgrip strength relative to body fat percentage with metabolic syndrome and its components.

Parameters	MetS	WC > 94 cm	Hypertension > 130/85 (mmHg)	Glucose > 100 (mg/dL)	HDL < 40 (mg/dL)	TG > 150 (mg/dL)
ROC (95%CI)						
HGS (kg)	0.54 (0.49–0.57)	0.49 (0.46–0.54)	0.53 (0.49–0.58)	0.54 (0.50–0.58)	0.48 (0.44–0.52)	0.54 (0.49–0.59)
HGS/BMI	0.66 (0.63–0.70) **	0.75 (0.72–0.79) **	0.61 (0.57–0.65) **	0.56 (0.52–0.60) **	0.54 (0.49–0.58)	0.62 (0.57–0.66) **
HGS/Weight	0.67 (0.63–0.71) **	0.79 (0.76–0.82) **	0.60 (0.56–0.64) **	0.55 (0.51–0.59) **	0.54 (0.49–0.58)	0.61 (0.57–0.65) **
HGS/%Fat	0.70 (0.65–0.72) **	0.80 (0.77–0.83) **	0.62 (0.58–0.66) **	0.56 (0.52–0.60) **	0.56 (0.52–0.60) **	0.63 (0.59–0.67) **

**Note:** Data are presented as receiver operating characteristic values (95% confidence interval). ** represent significant *p*-values at the 0.05 and 0.01 levels. HGS: handgrip strength; BMI: body mass index; %Fat: fat percentage; MetS: metabolic syndrome; WC: waist circumference; TG: triglycerides; HDL: high-density lipoprotein.

**Table 3:** Linear regression analysis for metabolic syndrome components with age, handgrip strength, and body composition.

Parameters	WC	SBP	DBP	Glucose	HDL	TG
Beta (95%CI)						
Model 1						
Adjusted R^2^	0.427	0.183	0.095	0.135	0.021	0.066
Age	0.22 (0.15–0.28) **	0.41 (0.35–0.48) **	0.15 (0.10–0.20) **	1.25 (1.01–1.47) **	−0.03 (−0.09–0.02)	1.23 (0.88–1.58) **
Weight	0.75 (0.69–0.82) **	0.15 (0.09–0.21) **	0.14 (0.09–0.19) **	−0.01 (−0.22–0.21)	−0.11 (−0.16 to −0.06) **	0.46 (0.14–0.78) **
HGS	−0.20 (−0.34 to −0.07) **	0.06 (−0.07–0.19)	0.12 (0.03–0.22) *	0.16 (−0.28–0.59)	0.04 (−0.06–0.14)	0.49 (−0.18–1.17)
Model 2						
Adjusted R^2^	0.408	0.191	0.101	0.135	0.021	0.073
Age	0.14 (0.07–0.21) **	0.39 (0.33–0.46) **	0.13 (0.08–0.18) **	1.23 (1.0–1.46) **	−0.03 (−0.08–0.03)	1.23 (0.88–1.58) **
BMI	2.28 (2.08–2.48) **	0.55 (0.36–0.74) **	0.47 (0.34–0.61) **	0.14 (−0.49–0.78)	−0.32 (−0.47 to −0.17) **	1.63 (0.64–2.61) **
HGS	0.02 (−0.12–0.15)	0.09 (−0.03–0.21)	0.16 (0.07–0.25)**	0.14 (−0.28–0.56)	0.02 (−0.08–0.12)	0.54 (−0.11–1.19)
Model 3						
Adjusted R^2^	0.398	0.189	0.087	0.136	0.041	0.088
Age	0.07 (0.01–0.15) *	0.38 (0.31–0.45) **	0.13 (0.07–0.18) **	1.27 (1.04–1.51) **	−0.01 (−0.06–0.05)	1.09 (0.73–1.45) **
%Fat	1.65 (1.5–1.79) **	0.38 (0.24–0.52) **	0.30 (0.20–0.39) **	−0.27 (−0.73–0.20)	−0.33 (−0.44 to −0.22) **	1.84 (1.12–2.54) **
HGS	0.27 (0.14–0.40) **	0.15 (0.03–0.27) *	0.21 (0.12–0.30) **	0.17 (−0.24–0.58)	−0.02 (−0.12–0.08)	0.70 (0.06–1.34) *

**Note:** Data are presented as beta (95% confidence interval), and adjusted R^2^ explains the variation in the model. * and ** represent significant *p*-values at the 0.05 and 0.01 levels. HGS: handgrip strength; BMI: body mass index; %Fat: fat percentage; MetS: metabolic syndrome; WC: waist circumference; TG: triglycerides; HDL: high-density lipoprotein.

**Table 4:** Odds ratios of each quartile of handgrip strength, handgrip strength relative to body mass index, handgrip strength relative to weight, and handgrip strength relative to body fat percentage for metabolic syndrome.

Variables	HGS		HGS/BMI		HGS/Weight		HGS/%Fat	
	OR (95% CI)	*p*-Value	OR (95% CI)	*p*-Value	OR (95% CI)	*p*-Value	OR (95% CI)	*p*-Value
**Q1**	1		1		1		1	
**Q2**	0.81 (0.54–1.21)	0.307	0.64 (0.44–0.94)	0.024	0.59 (0.41–0.86)	0.006	0.68 (0.5–0.99)	0.044
**Q3**	0.69 (0.45–1.06)	0.089	0.39 (0.27–0.58)	<0.001	0.37 (0.25–0.56)	<0.001	0.40 (0.3–0.60)	<0.001
**Q4**	0.72 (0.47–1.10)	0.132	0.23 (0.15–0.36)	<0.001	0.19 (0.13–0.31)	<0.001	0.15(0.10–0.25)	<0.001

**Note:** Data are presented as odds ratio (95% confidence interval). *p*-values less than the 0.05 and 0.01 levels are significant. HGS: handgrip strength; BMI: body mass index; %Fat: fat percentage.

**Table 5:** Odds ratio of each quartile of handgrip strength, handgrip strength relative to body mass index, handgrip strength relative to weight, and handgrip strength relative to body fat percentage for metabolic syndrome components.

Variables	High Waist		Hypertension		High Glucose		Low HDL		High Triglycerides	
	OR (95% CI)	*p*-Value	OR (95% CI)	*p*-Value	OR (95% CI)	*p*-Value	OR (95% CI)	*p*-Value	OR (95% CI)	*p*-Value
HGS										
Q1 Q2 Q3 Q4	1 0.7 (0.5–1.1) 0.8 (0.5–1.2) 1.1 (0.7–1.6)	0.09 0.19 0.92	1 0.6 (0.4–0.9) 0.4 (0.3–0.7) 0.8 (0.5–1.2)	0.03 <0.001 0.21	1 0.9 (0.6–1.4) 1.0 (0.6–1.4) 0.7 (0.4–0.9)	0.73 0.82 0.04	1 1.3 (0.9–1.9) 1.6 (1.1–2.4) 1.3 (0.8–1.9)	0.19 0.03 0.26	1 0.7 (0.5–1.2) 0.9 (0.6–1.4) 0.6 (0.4–1.1)	0.17 0.66 0.06
HGS/BMI										
Q1 Q2 Q3 Q4	1 0.9 (0.6–1.5) 0.23 (0.1–0.4) 0.13 (0.08–0.2)	0.71 <0.001 <0.001	1 0.7 (0.5–1.0) 0.5 (0.3–0.8) 0.4 (0.3–0.6)	0.06 0.001 <0.001	1 0.8 (0.6–1.2) 0.7 (0.5–1.1) 0.6 (0.4–0.9)	0.26 0.09 0.02	1 0.9 (0.6–1.4) 0.9 (0.6–1.3) 0.6 (0.4–0.9)	0.81 0.02	1 0.9 (0.6–1.3) 0.7 (0.4–1.0) 0.3 (0.2–0.5)	0.56 0.06 <0.001
HGS/Weight										
Q1 Q2 Q3 Q4	1 0.5 (0.3–0.8) 0.2 (0.1–0.3) 0.1 (0.04–0.1)	0.003 <0.001 <0.001	1 0.6 (0.4–0.9) 0.5 (0.3–0.7) 0.4 (0.3–0.6)	0.03 <0.001 <0.001	1 0.9 (0.7–1.4) 0.8 (0.5–1.2) 0.6 (0.4–0.9)	0.81 0.22 0.014	1 0.8 (0.6–1.2) 0.8 (0.6–1.2) 0.7 (0.5–1.1)	0.34 0.30 0.08	1 0.7 (0.5–1.1) 0.7 (0.4–1.0) 0.3 (0.2–0.5)	0.13 0.06 <0.001
HGS/%Fat										
Q1 Q2 Q3 Q4	1 0.8 (0.5–1.2) 0.2 (0.1–0.4) 0.06 (0.03–0.09)	0.26 <0.001 <0.001	1 0.7 (0.4–0.9) 0.6 (0.4–0.8) 0.3 (0.1–0.5)	0.042 <0.001 <0.001	1 1.0 (0.7–1.5) 0.7 (0.5–1.1) 0.6 (0.4–0.8)	0.93 0.12 0.004	1 0.8 (0.5–1.2) 1.0 (0.7–1.5) 0.5 (0.3–0.7)	0.32 0.97 0.001	1 0.7 (0.5–1.1) 0.8 (0.5–1.2) 0.2 (0.1–0.4)	0.12 0.23 <0.001

**Note:** Data are presented as odd ratios (95% confidence interval). *p*-values less than the 0.05 and 0.01 levels are significant. HGS: handgrip strength; BMI: body mass index; %Fat: fat percentage.

## Data Availability

All the data are available in the manuscript.
